# Changes in biosafety practices of Brazilian orthodontists after the COVID-19 pandemic: a cross-sectional study

**DOI:** 10.1590/1807-3107bor-2025.vol39.111

**Published:** 2025-11-07

**Authors:** Lucas Gonçalves SANTOS, Elio da Mata SANTOS JÚNIOR, Renata Pacífico de CARVALHO, Felipe Weidenbach DEGRAZIA, Rodrigo Hermont CANÇADO, José Fernando Castanha HENRIQUES, Daniela GARIB, Leniana Santos NEVES

**Affiliations:** (a) Universidade de São Paulo – USP, Bauru Dental School, Department of Orthodontics, Bauru, SP, Brazil.; (b) Universidade Federal de Minas Gerasis – UFMG , School of Dentistry, Department of Restorative Dentistry, Belo Horizonte, MG, Brazil.

**Keywords:** SARS-CoV-2, Orthodontists, Containment of Biohazards, Disinfection, Sterilization

## Abstract

The aim of this study was to assess the biosafety practices adopted during the COVID-19 pandemic that persisted in post-pandemic orthodontic clinical routines. In this cross-sectional study, 722 Brazilian orthodontists who were in the early phase of the pandemic, and 203 from the later phase, respectively, answered a 45-item questionnaire addressing basic personal information, use of personal protective equipment, biosafety protocols, and COVID-19 incidence. Descriptive statistics and chi-square tests were used to analyze the data. Most participants were infected with the coronavirus (from 10.1% to 65%). While 52.4% perceived providing care as very risky during the pandemic, this perception shifted to decreased to a moderate level of risk in the late phase of the pandemic (40.4%). The use of protective face masks/face shields decreased from 51.7% to 25.1%, as well as the use of disposable coats (from 77.1% to 45.8%). Many orthodontists discontinued the use of PFF2/N95 masks and reverted to wearing surgical masks again. Most orthodontists disinfected orthodontic bands, and photographic retractors through manual washing and autoclaving. Most participants preferred to clean their orthodontic pliers with 70% alcohol. A decline in orthodontists’ concern about biosafety during clinical appointments was observed in the late phase of the pandemic. Moreover, the need for greater specific care still persists, especially regarding the proper use of disposable coats, face shields, and surgical masks.

## Introduction

The emergence of SARS-CoV-2 and the subsequent declaration of a pandemic by the World Health Organization (WHO) in March 2020 have led to a notable increase in concerns regarding biosafety standards in clinical practice.^1^ Dentists, according to the North American Occupational Information Network, find themselves in the highest risk positions for disease transmission; consequently, they have to be more careful in their clinical practice.^2^

The use of personal protective equipment (PPE) is well established in contemporary dental practice. Nonetheless, guidelines, reference materials, and regulations issued by Brazilian institutions are still lacking. Nevertheless, previous studies conducted prior to the pandemic had already highlighted the non-compliance of Brazilian dentists with PPE standards established by healthcare authorities.^3-5^

Recently, Brazilian government entities such as the Federal Council of Dentistry and the National Health Surveillance Agency have issued recommendations for dental clinical practice during the SARS-CoV-2 pandemic, particularly focusing on precautions to be taken before and during treatment, including questionnaires to assess patients’ potential exposure to COVID-19 and instructions on the proper use of recommended PPE. In addition, previous studies available in the literature have already documented methods for controlling the contamination of materials routinely used in orthodontic clinical practice.^6,7^ Concurrently, although cleaning surfaces with 70% alcohol is highly effective in certain clinical scenarios,^8^ this method alone fails to meet all the biosafety criteria in a dental office environment.^2,9,10^

It is crucial to check the actual routine followed by Brazilian orthodontists in regard to biosafety measures adopted during the pandemic and to observe any changes in biosafety practices in orthodontic clinics after the pandemic. The aim of this study was to evaluate changes in biosafety practices by Brazilian orthodontists following the COVID-19 pandemic.

## Methods

This cross-sectional study was approved by the Research Ethics Committee of the School of Dentistry of the Federal University of Minas Gerais, and all patients signed an informed consent form before their participation in the study.

This study included Brazilian orthodontic professionals who voluntarily agreed to participate. A questionnaire containing 45 objective questions was applied at the beginning of the COVID-19 pandemic, in July 2020 and, in November 2022, an updated questionnaire containing the same questions was administered to orthodontic professionals in Brazil to assess their current clinician routines, their perception of biosafety practice and COVID-19 testing. All responses were collected anonymously, and it was not possible to identify whether the same individuals participated in both phases (T1 and T2), preventing direct response matching between time points.

The questionnaire was sent to the board of directors of the Brazilian Association of Orthodontics and Facial Orthopedics (ABOR), the Brazilian Association of Dentistry (ABO), and the São Paulo Society of Orthodontics (SPO). The Brazilian dental associations were contacted via email and requested to forward the questionnaire to their members, and approximately 4,000 emails were sent. The questionnaires were prepared using the Google Forms platform (Google, Mountainview, USA) and sent to dental associations, which redistributed them to their affiliated orthodontists through email or cell phone message via the WhatsApp Messenger (Facebook Inc., Menlo Park, USA).

The inclusion criteria consisted of dentists affiliated with ABOR, ABO, or SPO, specialized in orthodontics, who had valid email and telephone contact details and who voluntarily agreed to participate. No restrictions were imposed based on age, sex, Brazilian region, city of the professional, or time since graduation.

### Statistical analysis

The answers obtained from the questionnaires were analyzed using descriptive statistics and frequency analysis. The chi-square test was performed between some selected study variables.

Statistical analysis was carried out using Statistica for Windows (version 10.0; Statsoft, Tulsa, USA). All results with p<0.05 were considered statistically significant.

## Results

The study sample was obtained through convenience sampling via an online survey. A total of 772 orthodontists participated voluntarily in July 2020 and 203 orthodontists in November 2022, resulting in an overall response rate of 19.3% and 5.1%, respectively. Sample size was calculated to determine the minimum number of participants required, which was set at 271 based on an alpha of 0.05, an expected percentage of 22.8% among Brazilian orthodontists ^11^ (data collected from the Brazilian Federal Council of Dentistry), and a 95% confidence interval. The main demographic data and the major characteristics of biosafety practices of this cross-sectional study are summarized in Table 1.

Professionals from 24 out of the 26 Brazilian states, including the Federal District, participated in the study. A total of 62.4% of the participants were from the southeastern region of Brazil, 18.6% from the south, 9.3% from the northeast, 7.2% from the midwest, and 2.5% from the north. No professionals from the states of Acre or Roraima participated in our study. The mean ages of the participants in the early and late phases of the pandemic were 39.5 and 43.5 years, respectively, with a range of 24 to 65 years (Table 1). There was a predominance of women in both data extraction periods, with 66.3% in the early phase and 71.9% in the late phase (Table 1). Additionally, most orthodontists had more than 20 years of experience since graduation in both periods, with 43.8% in the early phase and 46.3% in the late phase of the pandemic. Most participants exclusively worked in private clinics, representing the primary service provided by orthodontists, accounting for 79.2% and 82.3% in the early and late phases, respectively (Table 1). When asked about the perceived risk of orthodontic care during the pandemic, 52.4% considered it to be of high risk. This percentage decreased to 40.4% in the late phase of the pandemic, indicating a moderate risk for orthodontic practice (Table 1).

Regarding COVID-19 testing, 25.5% of participants underwent COVID-19 tests during the early phase of the pandemic. This percentage significantly increased to 95.6% in the late phase (p < 0.05) (Tables 1 and 2). Among those who were tested, only 10.1% of orthodontists tested positive for coronavirus at the beginning of the pandemic (Table 1). However, this number increased to 65.0% in the late phase of data extraction (p < 0.05) (Tables 1 and 2).

During the early phase of the pandemic, 71.6% of the participants wore N95 or PFF2 masks during all clinical appointments. This percentage decreased to 50.3% in the late phase, and 37.4% of participants were no longer wearing N95 or PFF2 masks (Table 1). Hence, approximately half of the participants (51.7%) wore face masks or face shields during all clinical appointments in the first data extraction period. This percentage decreased further to 25.1% in the final period of the pandemic (Table 1). A significant percentage of participants (38.4%) were not currently wearing face masks/face shields. The use of disposable lab coats by orthodontists declined throughout the pandemic, from 77.1% to 45.8% (p < 0.05) (Tables 1 and 2). However, the use of protective goggles and disposable caps remained high throughout the pandemic, exceeding 70% and 80%, respectively (Table 1).

Disinfection and cleaning of materials routinely used in orthodontic clinics was also assessed, and the main findings are presented in Table 2. Most orthodontists initially disinfected orthodontic pliers by wiping them with 70% alcohol - 40.2% during the early phase of the pandemic and 54.7% in the late phase (Table 3). The disinfection of orthodontic pliers was carried out through manual washing (27.4%) and autoclaving (24.7%) (Table 3). When faced with the need to utilize photographic retractors for documenting their cases or include orthodontic bands into their treatment plans, most orthodontists in this study opted for manual washing and subsequent autoclaving of these instruments during the extraction data periods (Table 3). Also, wire markers were frequently used, especially in corrective orthodontic cases. When queried about the methods employed to clean wire markers, a significant percentage of over 63% of the participants indicated a preference for wiping them with 70% alcohol, a practice that remained consistent throughout both periods of the COVID-19 pandemic (Table 3).

**Table 2:** Comparison of answers between early and late pandemic phase (Chi-square tests)

Variable	Early pandemic phase n(%)	Late pandemic phase n(%)	p-value
COVID-19 testing			
Yes	198 (25.5)	194 (95.6)	< 0.05*
No	575 (74.5)	9 (4.4)	
COVID-19 results			
Positive	20 (10.1)	126 (65.0)	< 0.05*
Negative	178 (89.9)	68 (35.0)	
Disposable lab coat/disposable gown			
Yes	595 (77.1)	93 (45.8)	< 0.05*
No	177 (22.9)	110 (54.2)	
Protective/Safety goggles			
Yes	576 (74.7)	155 (76.4)	0.6099
No	196 (25.3)	48 (23.6)	
Disposable cap/disposable head cover			
Yes	670 (86.9)	169 (83.3)	0.1956
No	102 (13.1)	34 (16.7)	

*Statistically significant for p < 0.05.

**Table 3:** Disinfection and cleaning of materials routinely used in orthodontic practice.

Variable	Early pandemic phase n(%)	Late pandemic phase n(%)
Orthodontic pliers		
MW/UC/AP	70 (9.0)	5 (2.5)
MW/AP	212 (27.4)	50 (24.7)
MW only	14 (1.8)	10 (5.0)
AP only	11 (1.4)	4 (2.0)
Wiping with 70% alcohol	309 (40.2)	111 (54.7)
Other	153 (19.8)	23 (11.1)
Not used	3 (0.4)	0 (0)
Photographic retractors		
MW/UC/AP	95 (12.3)	38 (18.7)
MW/AP	267 (34.7)	93 (45.8)
MW only	14 (1.8)	5 (2.5)
AP only	19 (2.5)	7 (3.4)
Wiping with 70% alcohol	112 (14.4)	33 (16.3)
Other	27 (3.5)	5 (2.5)
Not used	238 (30.8)	22 (10.8)
Orthodontic bands		
MW/UC/AP	143 (18.3)	45 (22.2)
MW/AP	325 (42.2)	99 (48.7)
MW only	5 (0.7)	1 (0.5)
AP only	42 (5.5)	11 (5.4)
Wiping with 70% alcohol	29 (3.8)	16 (7.9)
Other	14 (1.8)	1 (0.5)
Not used	214 (27.7)	30 (14.8)
Wire markers		
MW/UC/AP	4 (0.6)	2 (1.0)
MW/AP	9 (1.20)	1 (0.50)
MW only	6 (0.80)	5 (2.5)
AP only	0 (0)	0 (0)
Wiping with 70% alcohol	489 (63.40)	145 (71.40)
Other	89 (11.50)	11 (5.40)
Not used	175 (22.50)	39 (19.20)

MW: Manual washing; UC: Ultrasonic cleaning; AP: Autoclaving process.

The results of the chi-square tests between the selected variables of this study are presented in Table 2.

## Discussion

This study stands out as one of the few investigations to examine the main changes in the utilization of PPE and the sterilization of orthodontic materials used on a daily basis among orthodontics professionals in Brazil over a 2-year period during the pandemic. These changes in the clinical routines of these professionals persisted even during the later pandemic period. The convenience sampling method yielded a noticeable discrepancy when comparing the obtained samples. Initially, 772 orthodontists voluntarily participated in the study, but this number decreased to 203 at the late pandemic phase. This decline can potentially be attributed to a limitation of scientific reports involving human experiments during the pandemic period. The presence of quarantine and lockdown measures imposed some restrictions on researchers and led to an increase in the number of reviews and questionnaire-based research studies conducted during the COVID-19 pandemic.^12-14^As a consequence, we hypothesize that the high demand for questionnaire-based research studies may have led to participant fatigue, reducing voluntary participation of Brazilian orthodontists in the final period of the pandemic.

Our findings demonstrate that most participants in our sample were women, with mean ages of 39.5 and 43.5 years during the two respective data collection periods. Previous studies have demonstrated a higher prevalence of female responses in studies involving questionnaires administered to health professionals.^15-17^These data are consistent with the latest survey published by the Brazilian Federal Council of Dentistry, which revealed that most of Brazilian orthodontists are women, with approximately 19,000 women and 12,000 men practicing in the field.^11^ Furthermore, this outcome was expected because recent studies involving questionnaires administered to health professionals have consistently shown a higher level of female participation.^16,18,19^ Regarding the healthcare setting in which participants work, the private sector predominated over the public sector. This finding may be related to the large number of orthodontics professionals in Brazil, which exceeds 30,000 specialists.^11^

A decrease in the perception of the risk of contamination has been observed during orthodontic appointments. Initially, orthodontists considered it to be a high risk during the early phase of the pandemic, but this perception shifted to a moderate level of risk in the late phase. This finding has been supported by a recent study conducted by Lima et al.,^19^ which showed that social isolation and quarantine measures resulted in higher levels of stress, anxiety, insomnia, and depression among Brazilian orthodontists at the start of the pandemic. Furthermore, the results of the study also demonstrate moderate to extreme financial impacts. On the other hand, with the introduction of new vaccines and the decline in COVID-19 mortality worldwide, psychological conditions such as stress and anxiety among health workers have decreased in recent years. Nevertheless, it is important for professionals to exercise caution as they still harbor concerns about exposing their friends and parents to the risk of infection.^15,20,21^

The present study revealed significant differences in COVID-19 tests and results between the two data extraction periods. After two years, most participants have been subjected to COVID tests (89.9%) and a significant percentage (p < 0.05) of participants (65%) tested positive for COVID. Additionally, there has been a decrease in the use of N95/PFF2 masks, face shields, and disposable lab coats. More than 600 million people have been infected by COVID-19, with healthcare professionals, particularly dentists, being at the forefront and facing a higher risk of contamination. Close contact with infected individuals remains the primary mode of transmission for this disease, which can spread through small respiratory droplets, such as saliva, released from the mouth or nose of an infected person.^22^ Even though the World Health Organization declared the end of the pandemic in May 2023, all healthcare professionals should exercise caution and sustain vigilance in their use of PPE because of the emergence of variants such as Omicron. This variant is particularly concerning as it exhibits increased transmissibility and a higher ability to evade immune responses compared to earlier strains of COVID-19, including Alpha, Beta, Gamma, and Delta.^22-24^

In assessing the disinfection practices of orthodontic materials, our findings demonstrate that orthodontists frequently employ manual washing and autoclaving for instruments such as photographic retractors and orthodontic bands. Most participants, however, preferred to clean their orthodontic pliers with 70% alcohol instead of using manual washing and autoclaving. In the field of orthodontics, numerous materials are frequently used in clinical practice; and orthodontists often have a high number of patient appointments per day. Therefore, it is concerning that 40.4% and 54.7% of the participants primarily clean their orthodontic pliers with 70% alcohol, especially considering that these instruments come into contact with human fluids such as saliva.^25^ It is known that cleaning surfaces with 70% alcohol effectively eliminates most microorganisms.^26^ However, spore-forming microorganisms, which rely on their ability to form spores as a survival strategy in adverse conditions, can persist on surface materials.^25,26^ For this reason, to prevent cross contamination, it is imperative for all Brazilian orthodontists to engage in manual washing and autoclaving of every orthodontic plier that comes into direct contact with a patient.

Although the present study encompassed participants from various Brazilian regions, the employment of a convenience sampling method should be acknowledged as a non-probability sampling technique. Consequently, the self-administration of the questionnaire introduces potential self-reporting biases. This possibility raises concerns regarding the external validity of the obtained results. Subsequent investigations should seek to validate these findings by employing a random sampling method. Also, the questionnaire did not ask about the vaccination of orthodontists because there were still no vaccines available in the first phase of questionnaire administration, and the intention was to use the same questions in both phases to allow for a comparison of changes in biosafety procedures. Future research endeavors should also consider the inclusion of qualitative interviews as a way of conducting research. These interviews would serve to shed light on various aspects of how orthodontists can enhance their instrument cleaning practices, as well as elucidate the reasons for the decrease in biosafety practices among Brazilian orthodontists.

### Clinical implications

The findings of this study highlight important shifts in biosafety protocols and risk perception among orthodontists throughout the COVID-19 pandemic. Initially, strict protective measures, such as the widespread use of N95/PFF2 masks, face shields, and disposable lab coats, were adopted to mitigate the risk of viral transmission. Conversely, as the pandemic progressed, adherence to these measures declined significantly, suggesting a shift in the perception of risk and possibly pandemic fatigue.^19^ This decline raises concerns about the long-term commitment of dental professionals to enhanced infection control measures, especially in light of potential future outbreaks of respiratory pathogens. Furthermore, while the initial implementation of strict biosafety protocols may have been driven by uncertainty and heightened caution, the eventual relaxation of these measures suggests the need for a more balanced and evidence-based approach to infection control in clinical settings.^21^

Additionally, the study underscores the impact of the pandemic on disinfection routines and the prevalence of infection among orthodontists. While the use of alcohol-based disinfection methods increased for certain instruments,^25^ other aspects of biosafety practices, such as the use of face shields and disposable protective clothing, have declined over time. This shift may have contributed to the substantial increase in the percentage of orthodontists testing positive for COVID-19 in the late phase of the pandemic, reinforcing the need for consistent adherence to infection prevention measures. These findings underscore the need for continuous education on biosafety protocols and the development of adaptable, long-term strategies for infection control in orthodontic practice. Maintaining a high standard of protective measures,^27^ even beyond immediate pandemic threats, is crucial for ensuring occupational safety and mitigating the transmission of infectious diseases in dental settings.

## Conclusion

Our findings have demonstrated a decrease in biosafety practices among Brazilian orthodontists, especially the reduction in the use of disposable lab coats, face masks/face shields, and N95/PFF2 masks in the late phase and after the pandemic. Implementation of strategies to increase awareness among Brazilian orthodontists regarding the significance of preventing cross-contamination in the clinical routine is necessary.

**Table 1:** Demographic characteristics and major changes in biosafety practices.

Variable	Early pandemic phase	Late pandemic phase
	n (%)	n (%)
Sex		
Female	512 (66.3)	148 (71.9)
Male	257 (33.3)	55 (28.1)
Prefer not to disclose	3 (0.4)	0 (0)
Age (years)	Mean (SD)	Mean (SD)
	39.5	43.5
	n (%)	n (%)
Time since graduation (years)		
< 5	80 (10.4)	14 (6.9)
5–10	128 (16.2)	42 (20.7)
10–20	226 (29.6)	53 (26.1)
> 20	338 (43.8)	94 (46.3)
Healthcare sector		
Only in public healthcare sector	20 (2.6)	9 (4.4)
Only in private healthcare sector	610 (79.2)	167 (82.3)
In both healthcare sectors	142 (18.2)	27 (13.3)
COVID-19 testing		
Yes	198 (25.5)	194 (95.6)
No	575 (74.5)	9 (4.4)
COVID-19 results		
Positive	20 (10.1)	126 (65.0)
Negative	178 (89.9)	68 (35.0)
Risky perception of care during the pandemic		
No risk	26 (3.4)	9 (4.5)
Low risk	140 (18.1)	48 (23.6)
Moderate risk	202 (26.1)	82 (40.4)
High risk	404 (52.4)	64 (31.5)
N95/PFF2 masks		
During all clinical appointments	552 (71.6)	102 (50.3)
Only in appointments involving aerosol generation	112 (14.6)	25 (12.3)
Not worn	108 (13.8)	76 (37.4)
Face shields		
During all clinical appointments	399 (51.7)	51 (25.1)
Only in appointments involving aerosol generation	274 (35.5)	74 (36.5)
Not worn	99 (12.8)	78 (38.4)
Disposable lab coat/disposable gown		
Yes	595 (77.1)	93 (45.8)
No	177 (22.9)	110 (54.2)
Protective/Safety goggles		
Yes	576 (74.7)	155 (76.4)
No	196 (25.3)	48 (23.6)
Disposable cap/disposable head cover		
Yes	670 (86.9)	169 (83.3)
No	102 (13.1)	34 (16.7)

## Data Availability

The authors declare that all data generated or analyzed during this study are included in this published article.
